# Preparation, Optimization and Toxicity Evaluation of (SPION-PLGA) ±PEG Nanoparticles Loaded with Gemcitabine as a Multifunctional Nanoparticle for Therapeutic and Diagnostic Applications

**Published:** 2017

**Authors:** Nima Hamzian, Maryam Hashemi, Mahdi Ghorbani, Mohammad Hossein Bahreyni Toosi, Mohammad Ramezani

**Affiliations:** a *Department of Medical Physics, School of Medicine, Shahid Sadoughi University of Medical Sciences, Yazd, Iran. *; b *Nanotechnology Research Center, School of Pharmacy, Mashhad University of Medical Sciences, Mashhad, Iran. *; c *Biomedical Engineering and Medical Physics Department, Faculty of Medicine, Shahid Beheshti University of Medical Sciences, Tehran, Iran. *; d *Medical Physics Research Center, Faculty of Medicine, Mashhad University of Medical Sciences, Mashhad, Iran. *; e *Pharmaceutical Research Center, School of Pharmacy, Mashhad University of Medical Sciences, Mashhad, Iran.*

**Keywords:** Gemcitabine, multifunctional nanoparticles, SPION-PLGA drag delivery system, optimized preparation parameters, human breast cancer cell line (MCF-7)

## Abstract

The aim of this study was to develop a novel multifunctional nanoparticle, which encapsulates SPION and Gemcitabine in PLGA ± PEG to form multifunctional drug delivery system. For this aim, super paramagnetic iron oxide nanoparticles (SPIONs) were simultaneously synthesized and encapsulated with Gemcitabine (Gem) in PLGA ± PEG copolymers via W/O/W double emulsification method. Optimum size and encapsulation efficiency for radiosensitization, hyperthermia and diagnostic applications were considered and the preparation parameters systematically were investigated and physicochemical characteristics of optimized nanoparticle were studied. Then SPION-PLGA and PLGA-Gem nanoparticles were prepared with the same optimized parameters and the toxicity of these nanoparticles was compared with Gemcitabine in human breast cancer cell line (MCF-7). The optimum preparation parameters were obtained with Gem/polymer equal to 0.04, SPION/polymer equal to 0.8 and 1% sucrose per 20 mg of polymer. The hydrodynamic diameters of all nanoparticles were under 200 nm. Encapsulation efficiency was adjusted between 13.2% to 16.1% for Gemcitabine and 48.2% to 50.1% for SPION. *In-vitro* Gemcitabine release kinetics had controlled behavior. Enhancement ratios for PLGA-Gem and SPION-PLGA-Gem at concentration of nanoparticles equal to IC50 of Gemcitabine were 1.53 and 1.89 respectively. The statistical difference was significant (*p*-value = 0.006 for SPION-PLGA-Gem and *p*-value = 0.015 for PLGA-Gem compared with Gemcitabine). In conclusion, we have successfully developed a Gemcitabine loaded super paramagnetic PLGA-Iron Oxide multifunctional drag delivery system. Future work includes *in-vitro* and *in-vivo* investigation of radiosensitization and other application of these nanoparticles.

## Introduction

Gemcitabine (2ʹ,2ʹ-difluoro-2ʹDeoxycytidine) as a chemotherapy agent is a deoxycytidine analog with similar structure to Cytarabine. Gemcitabine (Gem) as a single agent or in combination with other agents is effective for treatment of various solid tumors and a number of hematologic malignancies (1, 2). Gemcitabine has chemical instability, poor cell uptake and a very short half-life in plasma. These properties lead to use of higher doses and result in systemic toxicity which is known as a side effect of this chemotherapy drug (3, 4).

Magnetic nanoparticles have been investigated as carriers for targeted drug delivery to overcome this great disadvantage (5-8). This is due to their high magnetic responsiveness, biodegradability, biocompatibility, high delivery efficiency and potential targeting function (9-11). Moreover, iron oxide nanoparticles are the only magnetic nanoparticles approved for clinical application by the US Food and Drug Administration (FDA) (12). In a number of studies, Super Paramagnetic Iron Oxide Nanoparticles (SPIONs) have been used for diagnostic and therapeutic applications such as magnetic resonance imaging (MRI) by contrast agents (13), magnetic hyperthermia in cancer treatment (14) and radiosensitization in radiotherapy (15). In addition, drugs can be loaded onto magnetic nanoparticles for targeted therapy with the aid of an external magnet (5-7). For this purpose, magnetic Fe_3_O_4_ nanoparticles are firstly prepared and then are encapsulated in copolymers. 

Recently, the use of biodegradable copolymers of poly lactic-co-glycolic acid (PLGA) for delivery of chemotherapy drugs has increased the efficiency in treatment of tumors in a wide range of cancer types (3, 4). PLGA has been approved by FDA for human use (16). The advantages of this polymer are biodegradability, biocompatibility, ease of accumulation within the tumor, rapid clearance from biological systems and high efficiency of drug delivery (17-19). PLGA is strongly hydrophobic, which causes limitations in its use in practical drug formulations. Poly (ethylene glycol) (PEG) is a non-toxic, water-soluble, and biocompatible polymer. Block copolymers consisting of hydrophobic polyester and a hydrophilic PEG segment have gained increasing attention due to their biodegradability, biocompatibility, and tailor-made properties (20-23). Previous studies have shown that nanoparticles made of PEG-PLGA exhibit long-circulation properties*, in-vivo*. This property is because the surface of hydrophilic molecular chains of PEG cannot be recognized and phagocytized by mono-nuclear phagocyte systems (20, 21).

The aim of this study is to develop a novel drug delivery system in which Gemcitabine and SPION are encapsulated in PLGA ± PEG to form a multifunctional nanoparticle. For this purpose, firstly the incorporation of SPION and Gemcitabine into PLGA ± PEG nanoparticles was optimized via W/O/W double emulsification method. The preparation parameters of (SPION-PLGA-Gem) ± PEG were systematically investigated. 

Therefore, using the optimized formulation, nanoparticles could be produced with several expected characteristics, such as optimum encapsulation efficiency and size, with the aim of radiosensitization, hyperthermia and diagnostic applications. Then SPION-PLGA and PLGA-Gem nanoparticles were prepared with the same preparation method of the SPION-PLGA-Gem and physicochemical properties and *in-vitro* cytotoxicity of the nanoparticles was characterized on MCF7 human breast cancer cell line.

## Experimental

*Materials*

Ferric chloride hexahydrate (FeCl_3_- 6H_2_O, 98%), ferrous chloride tetrahydrate (FeCl_2_- 4H_2_O), and ammonium hydroxide (25% w.t) were purchased from Fluka (Buchs, Switzerland). Poly(d,l-lactic-co-glycolic acid) (PLGA) (Average Mw: 17,000-35,000; lactic acid: glycolic acid = 50:50), Poly (ethylene glycol) (PEG) (Average Mw: 9000) and 3-(4,5-Dimethylthiazol-2-yl)-2,5-diphenyltetrazolium bromide (MTT) were obtained from Sigma-Aldrich (Munich, Germany). Gemcitabine hydrochloride was purchased from Euroasia Co., Ltd. (Delhi, India). Roswell Park Memorial Institute (RPMI) 1640 medium, fetal bovine serum (FBS) and trypsin were purchased from GIBCO (Gaithersburg, USA Darmstadt, Germany). Polyvinyl alcohol (PVA, 87-89% hydrolyzed, average MW = 88,000-97,000) and other solvent and chemical agents were procured from Merck (Germany) without further purification. Water used in the experiments was deionized and ﬁltered (Milli-Q Academic, Millipore, France).

*Synthesis of SPIONs*

SPIONs were synthesized using a co-precipitation method (24). Briefly, 450 mL of deionized water was stirred mechanically for 15 min under a nitrogen gas at room temperature to remove O_2_ from solution. Then 0.19 mg FeCl_2_ (4H_2_O) and 0.486 mg FeCl_3_ (6H_2_O) were added to the vigorously stirred water and 250 mg of oleic acid was quickly added to the previous reaction mixture and the product container was placed in a water bath (75-80 ˚C). After 15 min, 1.35 mL of NH_4_OH was added during one minute and argon gas flow was discontinued. After about 30 min, SPIONs were deposited. The product was washed three times with deionized water and the black precipitate was separated using a permanent magnet and lyophilized.

*Preparation of (SPION- PLGA-Gem) ± PEG nanoparticles*

Double emulsion method (W_1_/O/W_2_) was used for preparation of SPION-PLGA-Gem nanoparticles. Briefly, 1 mL of SPION suspension in chloroform was mixed with 1 mL of the organic solution of the polymer (PLGA ± PEG) in dichloromethane. Then, 0.2 mL solution of Gem in deionized water was added to the organic phase, and the mixture was emulsiﬁed by probe sonication (Fisons Instruments Ltd,. Crawley, UK) for 1 min (0.6 Hz frequency, 90 amplitude) (W_1_/O). The primary water-in-oil (W/O) emulsion was added drop wise to 8 mL of ice-cold aqueous PVA solution (5%, W/V) and emulsified for 10 min using a probe sonicator (W_1_/O/W_2_). To evaporate the organic solvent, the resulted solution was diluted in 10 mL aqueous PVA solution (0.1%, w/v) under stirring at room temperature overnight. Then the nanoparticles were collected by centrifugation at 14000 rpm for 15 min and washed three times with deionized water. Finally, the products were freeze-dried and the dry samples were ﬁlled with N_2_ gas and stored in a freezer for further use.

In this study, the effect of various processing parameters on the mean diameter of nanoparticles or/and encapsulation efficiency (EE) were evaluated. These parameters included amount of sucrose as cryoprotector (0%, 1% and 3% per 20 mg of polymer), amount of PEG (0% or 20% by weight of the polymer), concentration of SPION (2, 4 and 10 mg per 1 mL of chloroform) and concentration of Gemcitabine hydrochloride (1 or 2 mg per 200 μL of deionized water, equivalent to Gem/Polymer of 0.04 and 0.08). The technical parameters of the preparation and the volume fractions were selected based on previous experiences in this field. The amount of polymer (PLGA ± PEG) was kept constant (25 mg per 1 mL of dichloromethane). 

*Preparation of SPION-PLGA, PLGA-Gem and PLGA nanoparticles*

The PLGA-Gem nanoparticles were prepared using similar method to that used for SPION-PLGA-Gem nanoparticles however there was no SPION in the oil phase. Furthermore SPION-PLGA nanoparticles were prepared with the same method except that there was no Gemcitabine in W_1_ phase.

For the evaluation of toxicity, PLGA nanoparticles were prepared in the same manner as the control group.

*Nanoparticles characterization*

*Loading content and encapsulation efficiency*

Loading and encapsulation efﬁciency of Gemcitabine were determined in triplicate using a UV-Vis spectrophotometer (Shimatzu, Tokyo, Japan) by an excitation wavelength, *λ *= 268 nm.

Regarding SPION-PLGA-Gem formulation, since the SPION has absorption in this range of wavelengths; the SPION should be removed from SPION-PLGA-Gem formulation. For this purpose, 5 mg of SPION- PLGA-Gem nanoparticles was dissolved in 1 mL of acetonitrile and mixed with 4 mL PBS (pH 7.4). The mixture was incubated for 30 min at room temperature and then centrifuged at 14,000 rpm for 20 min. The concentration of Gemcitabine in supernatant was determined by the UV-Vis spectrophotometer. The accuracy of separation was confirmed by comparing the absorptions of solution at wavelengths of 268 and 320 nm. For PLGA-Gem nanoparticles, 1 mg NPs was dissolved in 1 mL dimethylsulfoxide (DMSO) and the concentration of Gemcitabine in solution was analyzed using UV–visible spectrophotometer at 268 nm.

Loading contents and encapsulation efficiencies were calculated using the following formulas:

Loading contents (%) = (Drug weight in the nanoparticles /Weight of nanoparticles) × 100 

Encapsulation Efficiency (%) = (Residual drug in the nanoparticle /Initial feeding amount of drug) × 100 

Loading content and encapsulation efﬁciency of SPIONs were determined in triplicate using Atomic Absorption Spectrophotometer (AAS) (CTA-3000, ChemTech , UK) . HCl was used as a blank.

*Size distribution and zeta potential*

Hydrodynamic diameter, Poly Disparity Index (PDI), zeta potential and size distribution profile of nanoparticles were obtained with dynamic light scattering method. 1 mg of each polymeric formulation was dispersed in 1 mL of deionized water with bath sonication and analyzed with zetasizer (NANO-ZS, Malvern, UK). For SPIONs, 5 mg of nanoparticles were dispersed in 1 mL of chloroform.

*Transmission electron microscopy*

The particle size and morphology of the magnetic nanoparticles were observed by transmission electron microscopy (LEO 910, Zeiss, Germany). Lyophilized nanoparticles of SPION-PLGA-Gem were dispersed in ultrapure water with bath sonicator and SPIONs were dispersed in chloroform. The samples were deposited onto a copper grid (300 mesh) and left until the excess liquid was removed and then were dried under infrared light. The acceleration voltage was set to 100 kV for SPION and 80 kV for SPION-PLGA-Gem nanoparticles.

*Atomic force microscopy*

The surface morphology of SPION-PLGA-Gem nanoparticles was observed by Atomic Force Microscopy (AFM, model: Nano Wizard II NanoScience AFM, JPK Instruments Inc., Germany). The glass slide of microscope was washed with acetone and ethanol, and then was rinsed with ultrapure water and was dried. After cleaning, lyophilized nanoparticles were dispersed in ultrapure water with bath sonicator (0.1 mg/mL) and then were dispensed onto glass slide and left at room temperature until dried. The measurements were performed in the tapping mode and dehydrated state in air, and ACT cantilevers were used.

*In-vitro Gemcitabine release*

*In-vitro* release behavior of Gemcitabine from SPION-PLGA-Gem formulation was assessed in sink condition. 5 mg of formulation was reconstituted in 5 mL PBS (pH 7.4) and the solvent was placed at a hybridization oven (Brunswick Scientific CO., Inc., New Jersey, USA) at 37 ˚C, 25 rpm. At specified time intervals (2, 4, 8, and 12 h; 1, 2, 4 and 7 days), the sample was centrifuged (10 min, 14000 rpm) and 200 µL of supernatant was displaced with PBS. Then, the accumulative ratios of the released Gemcitabine were calculated as a function of time. The concentration of Gemcitabine in each sample was determined using UV-Vis spectrophotometer. 

*Cell line and cell culture*

MCF-7 Breast cancer cell line (obtained from Pasteur Institute of Iran) was cultured in the RPMI medium supplemented with heat-inactivated fetal bovine serum (FBS) (10% vol/vol), penicillin (100 U/mL), and streptomycin (100 µg/mL). The cultures were incubated at 37 °C in a humidified atmosphere containing 5% CO_2_ (vol/vol). For experiments, the cells were used while they were in the exponential growth phase.

*Toxicity of Gemcitabine and Nanoparticles*

For *in-vitro* evaluation of toxicity of Gemcitabine and prepared formulations, MTT assay was used. Briefly, MCF-7 cells were seeded into 96-well plates at density of 1 × 10^4^ cells/well. After 48 h incubation, different concentrations of Gemcitabine (0.01-25 µM) and their equivalent concentrations of the formulations (PLGA, SPION-PLGA, PLGA-Gem and SPION-PLGA-Gem) were dispersed in RPMI. Then the RPMI in each well was precisely replaced with the RPMI including the formulations. After 48-hours incubation, 20 µL MTT reagents in PBS (5 mg/mL) were added to each well and were incubated for 3.5 h at 37 ˚C. Then the medium was replaced with 100 µL of DMSO and the absorbance was measured with an ELISA microplate reader (TECAN inﬁnite M200, Switzerland) at 570/630 nm wavelength. The percentage of viability (100×absorbance of test/absorbance of control) was calculated for each group. The viability (%) of the cells under treatment by Gemcitabine was also studied using 24 h MTT assays.

*Statistical analysis*

All the data were obtained using SPSS 16.0 statistical package. The results were reported as mean ± standard deviation (SD) of triplicates. Statistical differences for multiple groups were obtained using a one-way ANOVA and individual groups were compared with Student’s t-test. Probabilities of P < 0.05 were considered as signiﬁcant.

## Results and discussion

Recently, there is much attention dedicated to develop and characterize of materials at nanoscale for various and new applications. In this study, SPION and Gemcitabine were simultaneously encapsulated in PLGA ± PEG to form a multifunctional drug delivery system. Optimum size and encapsulation efficiency were considered with respect to the radiosensitization, hyperthermia, and diagnostic applications as well as the preparation parameters were investigated systematically. Additional to the above issues, physicochemical characteristics of the optimized nanoparticles were studied. Then SPION-PLGA and PLGA-Gem nanoparticles were prepared with the same optimized parameters and the toxicity of these nanoparticles was compared with Gemcitabine alone in human breast cancer cell line (MCF-7).

*Optimization of preparation parameters of polymeric nanoparticles*

In this study, for optimization of preparation parameters of nanoparticles, various applications such as hyperthermia (with ultrasound or RF waves), sonoporation, radiosensitization in radiotherapy and contrast enhancement of ultrasound and MR images were considered. 

For final selection of the optimal preparation parameter of (SPION- PLGA-Gem) ± PEG nanoparticles, size and encapsulated efficiency (EE) were considered and the examined variables were the volume percentage of sucrose (as cryoprotectant); and weight fractions of Gemcitabine and SPION to the polymer. Moreover, the presence and absence of PEG was examined in order to improve the physicochemical properties of nanoparticles for in*-vivo* studies. 

For selection of volume fractions, sonication time, washing and other parameters, the previous studies and experiments were taken into account.

Then, for better comparison of various formulations, weight fractions for (PLGA-Gem) ± PEG, (SPION-PLGA) ± PEG and PLGA ± PEG nanoparticles were selected similar to the (SPION-PLGA-Gem) ± PEG nanoparticles.

*Optimization of formulation with sucrose*

In order to evaluate the effect of sucrose in nanoparticles size, 0%, 1% and 3% sucrose per 20 mg of PLGA ± PEG were examined. As it is evident from the data in Table 1. 3% sucrose has considerable effect on the optimization of nanoparticle size. Additionally, there has no considerable deference in nanoparticle size between the 1% and 3% sucrose. Therefore, for evaluation of the other parameters, 1% sucrose per 20 mg of polymer was used to increase the weight fraction of nanoparticles in the final product.

*Optimization of formulation with concentration of SPION*

To optimize the formulation with SPION, different concentrations of SPION (2, 4 and 10 mg per 1 mL chloroform, equivalent with 0.08, 0.16 and 0.4 of SPION/PLGA) were used. From the data presented in Table 2. it can be seen that although the size of the nanoparticles at SPION concentration of 4 mg/mL is higher than 2 mg /mL, but both sizes of nanoparticles are in the desirable range (about 200 nm), and thus the concentration of 2 mg/mL was neglected. Additionally, since the loading content and encapsulation efficiency of SPION and Gem in concentration of 10 mg/mL is better than 4 mg/mL, the optimum concentration is 10 mg/mL

*Optimization of formulation with weight ratio of Gem/polymer*

For this purpose, two fractions of 0.04 and 0.08 were examined. Nanoparticles that were characterized in Table 3. were prepared at fixed conditions of 1% sucrose, 10 mg/mL of SPION/chloroform. As it can be seen in Table 3. weight fractions of 0.04 and 0.08 had no considerable effect on nanoparticle size and encapsulation efficiency, therefore to improve the loading content of Gemcitabine, the fraction of 0.04 was selected.

*Addition of PEG to formulation*

To evaluate the effect of PEG, in all the experiments, 20% of PLGA was replaced with PEG. According to the results that were listed in Table 2 and Table 3. The existence of PEG has no considerable effect on the size and encapsulation efficiency of nanoparticles. 

*Optimized parameters for preparation of SPION-PLGA-Gem nanoparticles*

With respect to the previous results, for the SPION-PLGA-Gem formulation, the selected parameters included 25 mg of PLGA ± PEG in 1 mL dichloromethane, 1 mg Gem per 200 mL of deionized water (equivalent to Gem/polymer of 0.04), 10 mg SPION per 1 mL of chloroform (equivalent to SPION/polymer of 0.4) and 1% sucrose per 20 mg of PLGA ± PEG.As it was mentioned before, nanoparticles made from PEG-PLGA exhibit *in-vivo* long-circulation properties (20, 21). Therefore, for examination of *in-vitro* toxicity of the prepared nanoparticles, PEG was not used. 

**Table 1:** Hydrodynamic diameter (*Z*_ave_) and PDI of (PLGA-Gem-SPION) ± PEG nanoparticles obtained by zetasizer. The sucrose was considered as the dependent variable

PDI (Mean)	${\mathrm{Size}}_{\mathrm{ave}}$ (nm)^*^ (Mean ± SD)	Sucrose (%)	PEG (mg)	SPION )mg/mL)	PLGA (mg)	$\frac{m_{\mathrm{Gem}}}{m_{\mathrm{Polymer}}}$
0.23	89.0±1044.0	0	5	10	20	0.08
0.17	178.5±9.6	1	5	10	20	0.08
0.15	8.3±170.2	3	5	10	20	0.08
0.15	66.6±678.5	0	0	10	25	0.08
0.09	173.4±0.5	1	0	10	25	0.08
0.05	3.1±171.2	3	0	10	25	0.08

**Table 2:** Hydrodynamic diameter (Z_ave_), PDI and encapsulation efficiency of SPION and Gemcitabine in (PLGA-Gem-SPION) ± PEG nanoparticles. The SPION and PEG were considered as dependent variables. Z_ave _and PDI were obtained by zetasizer

PDI* Mean	* ${\mathrm{Size}}_{\mathrm{ave}}(\mathrm{nm})$ Mean±SD	%EE (Gem) Mean±SD	%EE(SPION)Ŧ Mean±SD	SPION )mg/mL)	$\frac{m_{\mathrm{Gem}}}{m_{\mathrm{Polymer}}}$	PLGA	PEG	Sucrose (%)
0.17	178.5±9.6	17.1±0.4	0.6±40.2	10	0.08	20	5	1
0.09	173.4±0.5	13.8±0.3	3.4±44.9	10	0.08	25	0	1
0.15	160.0±4.1	15.2±1.3	3.2±45.1	4	0.08	20	5	1
0.10	163.2±1.2	11.6±0.9	5.1±47.3	4	0.08	25	0	1
0.15	5.2±165.3	15.2±1.6	3.1±45.1	2	0.08	20	5	1
0.10	3.4±161.1	11.6±2.3	5.1±47.3	2	0.08	25	0	1

* Obtained by UV-Vis spectrophotometer.

Ŧ Obtained by atomic absorption spectrophotometer

**Table 3:** Hydrodynamic diameter (Z_ave_), PDI and encapsulation efficiency of SPION and Gemcitabine in (PLGA-Gem-SPION) ± PEG nanoparticles. The Gemcitabine ratio and PEG were considered as dependent variables. Z_ave _and PDI were obtained by zetasizer

PDI* Mean	* ${\mathrm{Size}}_{\mathrm{ave}}(\mathrm{nm})$ Mean±SD	%EE (Gem) Mean±SD	%EE(SPION)Ŧ Mean±SD	SPION )mg/mL)	$\frac{m_{\mathrm{Gem}}}{m_{\mathrm{Polymer}}}$	PLGA	PEG	Sucrose (%)
0.08	8.0±174.0	16.1±2.0	47.7±2.6	10	0.04	20	5	1
0.17	178.5±9.6	17.1±0.4	0.6±40.1	10	0.08	20	0	1
0.08	10.0±180.0	16.0±1.0	48.2±2.1	10	0.04	25	5	1
0.09	173.4±0.5	13.8±0.3	3.4±44.9	10	0.08	25	0	1

* Obtained by UV-Vis Spectrophotometer.

Ŧ Obtained by atomic absorption spectrophotometer

**Table 4:** Size, zeta potential and PDI of nanoparticles with optimum preparation parameters

**Formulation**	***Z***_ave_ **(nm)** ^**^ **(mean±SD)**	**Size (nm)** **(TEM)**	**PDI** * **(mean±SD)**	**Zeta potential (mV)** **(mean±SD)**
SPION	2.2±20.1	7.0±0.5	0.20±0.05	+20.2±2.0
PLGA	190.6±8.6	-	0.03±0.01	-15.3±0.5
SPION- PLGA	170.3±4.6	-	0.16±0.04	-12.1±1.0
PLGA-Gem	175.2±8.3	-	0.05±0.02	-14.2±1.1
SPION-PLGA-Gem	180.2±10.3	180.0±20.0	0.08±0.02	-13.3±1.0

* Poly Disperse Index (PDI), *Z*_ave_ and zeta potential (mV) were obtained by DLS.

**Table 5:** Hydrodynamic diameter (nm) of polymeric nanoparticles (by DLS) and average size (nm) of SPION (by TEM) in this study compared with previous studies

**Formulation**	**This study** **(mean ± SD)**	**Other studies** *	**References**
SPION (TEM)	7.0±0.5	4-20	(37-41)
PLGA-SPION	170.0±4.6	293-110	(38, 39, 42)
PLGA-Drug	-	50-190	(18, 43)
PLGA-Gem	175.0±8.3	132-206	(1, 3, 36, 37)
SPION-PLGA-Drug	180.0±10.0	200-300	(40, 44)

* Other studies with approximately similar synthesis and preparation methods

**Table 6:** Loading content and encapsulation efficiency of Gem and (or) SPION in formulations. Encapsulation efﬁciency of SPION and Gemcitabine were determined in triplicate using atomic absorption spectrophotometer and UV-Vis spectrophotometer, respectively

**Formulation**	**Encapsulation efficiency (%)**		**Loading content** **(%)**	
	**Gem( UV-Vis)**	**SPION(AAS)**	**Gem**	**SPION**
SPION-PLGA	-	50.1±1.1	-	28.6
PLGA-Gem	13.2±1.3	-	3. 8	-
SPION-PLGA-Gem	16.1±2.2	48.2±2.1	2.8	27.8

**Table 7:** Enhancement ratios and survival (%) of MCF-7 cells in various treatment groups after 48 h incubation. The concentration of formulations is equivalent to IC50 of Gemcitabine (10.3 µM

**Formulation**	**Survival (%)**	**Enhancement ratio**	***P*** **-value** *
PLGA-SPION	93.1±1.2	**-**	**-**
Gem	50.0±0.0	1.00	-
PLGA-Gem	32.7±1.1	1.53	0.015
SPION-PLGA-Gem	26.2±2.3	1.89	0.006

* *P*-values are calculated compared to Gemcitabine group

**Figure 1:**
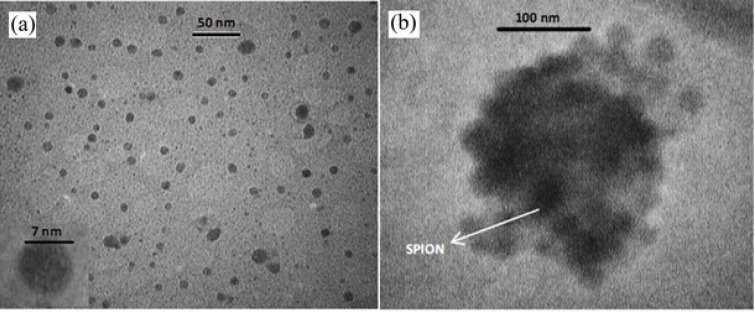
TEM image of SPIO nanoparticles (a), TEM image of SPION-PLGA-Gem nanoparticles (b).The samples were deposited onto a copper grid (300 meshes). The acceleration voltage was set to 100 kV for SPION and 80 kV for SPION-PLGA-Gem nanoparticles

**Figure 2:**
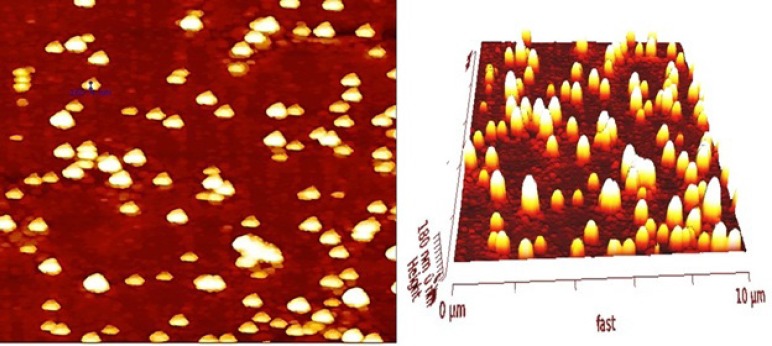
AFM image of SPION-PLGA-Gem nanoparticles. Lyophilized nanoparticles (100 µg) were dispersed in ultrapure water with bath sonicator (0.1 mg/ml) and were dispensed onto the glass slide of microscope

**Figure 3:**
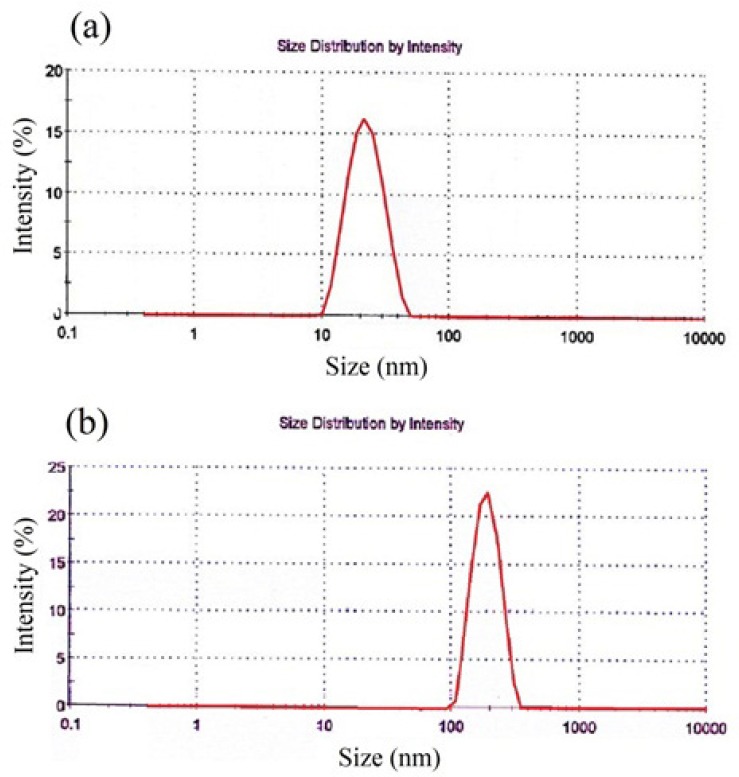
Size distribution of nanoparticles obtained by AFM, SPIONs (a), SPION-PLGA-Gem nanoparticles (b

**Figure 4:**
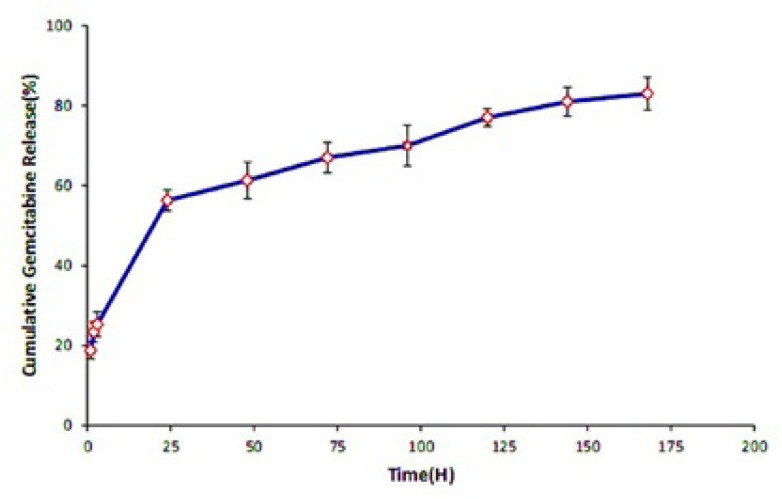
*In-vitro* cumulative release of Gemcitabine from SPION-PLGA-Gem nanoparticles at the specified intervals (2, 4, 8, 12 hours; 1, 2, 4 and 7 days) in PBS (7.4 pH). The data were obtained by UV-Vis spectrophotometry

**Figure 5:**
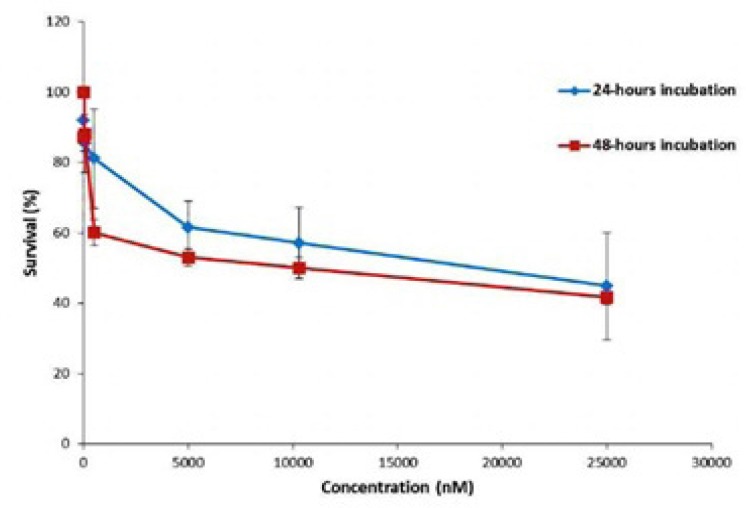
Survival (%) of MCF-7 cells treated by Gemcitabine after 24 and 48-hours incubation. The serial concentrations of Gemcitabine are 0.01-25 µM

**Figure 6:**
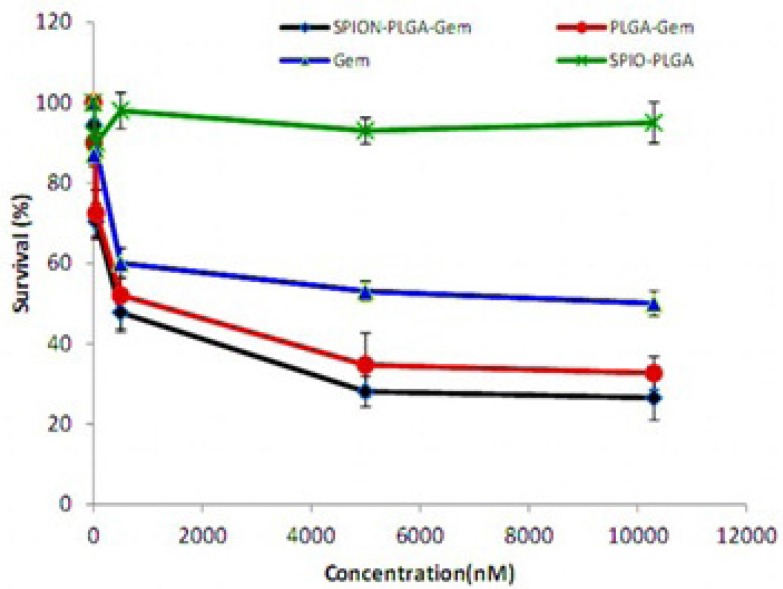
Survival (%) of MCF-7 cells of various treatment groups after 48-hours incubation. The concentrations of formulations are equivalent the concentration of 0.01 µM to 10.3 µM of Gemcitabine

**Figure 7:**
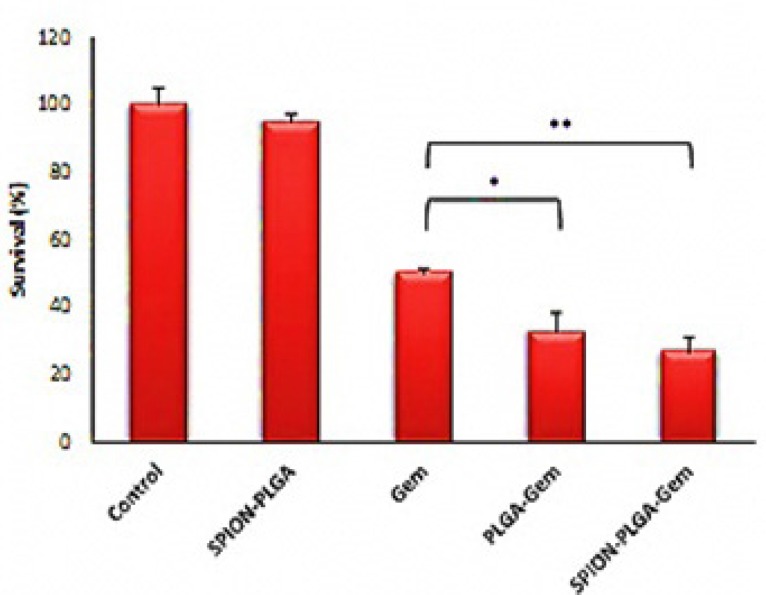
Survival (%) of MCF-7 cells of various treatment groups after 48-hours incubation. The concentration of formulations is equivalent to IC50 of Gemcitabine (10.3 µM). *p≤0.05 compared to Gemcitabine group, **p≤0.01 compared to Gemcitabine group

*Physicochemical properties of nanoparticles*

*Morphology and size distribution*

Morphology of prepared nanoparticles obtained by TEM and AFM in Figure 1 and 2. illustrates that SPIONs prepared by chemical coprecipitation method are spherical with uniform distribution and SPIO-PLGA-Gem nanoparticles prepared by a double emulsion method are also have nearly spherical shape and is uniformly distributed. Entrapped SPIONs in these nanoparticles are obvious as dark spherical points. 

*Size, size distribution and zeta potential*

Hydrodynamic diameter, Poly Disparity Index (PDI), zeta potential and size distribution profile of nanoparticles were obtained by dynamic light scattering (DLS) method. According to Table 4. the average hydrodynamic diameter of these nanoparticles is 20.1 ± 2.2 nm with PDI of 0.20 ± 0.05. The size of SPIONs that was obtained by TEM is 7.0 ± 0.5 nm. Zeta potential of these nanoparticles is 20.2 ± 2.0 mV. These nanoparticles have narrow size distribution (Figure 3 (a)). 

As listed in Table 4. the average size of SPION-PLGA-Gem nanoparticles that was obtained by TEM is 180.0 ± 20.0 nm. The average hydrodynamic diameter of these nanoparticles obtained by DLS is 180.2 ± 10.3 nm with PDI of 0.08 ± 0.02. Zeta potential of these nanoparticles is -13.3 ± 1.0 mV. SPION-PLGA-Gem nanoparticles disperses easily in water and be collected by permanent magnet. As it illustrated in Figure 3 (b). the size distribution of SPION-PLGA-Gem nanoparticles is Gaussian, narrow, and symmetrical.From the data listed in Table 4. the average Hydrodynamic diameters of polymeric nanoparticles with minor differences were less than 200 nm. The size of the PLGA nanoparticles (without drug and SPION), is slightly more than all other provided nanoparticles (190 ± 8.6). This indicates that the existence of SPION in the formulation, on the one hand, increases PDI and on the other hand, may not have a considerable impact on the size and make slight adjustments to it. This may be due to the role of the SPION nanoparticles in stability and prevention of aggregation of polymeric nanoparticles. 

In Table 5. the sizes of prepared nanoparticles are compared with the sizes of the same nanoparticles provided in the other studies. As it can be seen, the sizes of these nanoparticles are in an appropriate range, compared with the other studies using similar preparation methods.

*Drug loading and entrapment efficiency*

As can be seen from Table 6. the SPION encapsulation efficiency, using AAS method, is about 50%. Of course this amount is affected by the loading content of SPIONs in the polymeric nanoparticles. SPION loading content in provided polymeric nanoparticles is about 28%. Gemcitabine loading capacity in polymeric nanoparticles is between 2.8% for the SPION-PLGA-Gem formulation and 3.8% for the PLGA-Gem formulation. 

Efficiency of Gemcitabine encapsulation is 16.1 ± 2.2 (%) and 13.2 ± 1.3 (%) in these formulations, respectively. 

Higher performance of SPION encapsulation (4 folds) in SPION-PLGA-Gem formulation compared to Gemcitabine may be effective for radiosensitivity and imaging purposes. The cause of the issue is that in these applications, concentrations of Gemcitabine with minimal toxicity are used, while considering the lower toxicity of SPION compared to Gemcitabine, there is no special restrictions on the use of SPION compared with Gemcitabine. Using higher density of SPION leads to the effectiveness of diagnosis and treatment. 

Therefore, this amount of the difference in Encapsulation Efficiency between SPION and Gemcitabine appears to be favorable for application purposes. 

*In-vitro release of Gemcitabine*

Due to chemical instability and poor cell harvest, Gemcitabine has a very short half-life in plasma after intravenous injection (25). Therefore, it should be used with high doses leading to high systemic toxicity (1, 3). To solve this problem, in the past decades, nanotechnology has an improvement, and has emerged as a basis for the treatment of a wide range of different tumors. Nanotechnology leads to a prolongation of the release and increasing the entrance of drug into the cell (1). Nano platforms increase anti-tumor effects with negligible toxicity and causes controlled transfer and accumulation in tumor area and protection of drug molecules against biodegradability and plasma clearance (2).

In this study, PLGA nanoplatforms were used. The advantages of this polymer could be biodegradability, biocompatibility, decreasing their systemic side effects, facilitating intratumoral encapsulation, rapid clearance from the biological system and high efficiency of drugs transmission and transportation (17, 18, 26). Therefore, they have been used in many micro-formulations and nanoparticles (27-30). For example, fluorouracil (31), doxorubicin (7), docetaxel (32, 33), paclitaxel (34, 35) and cisplatin (20) could be mentioned. 

Following the physicochemical characterization of NPs, *in-vitro* Gemcitabine release profile from SPION-PLGA-Gem NPs has been conducted in a PBS solution at pH 7.4. As illustrated in Figure 4. it can be seen that in the first 24 h, release rate grows with a slight slope and reaches more than 60% after 48 h. After this time the growth of the curve is very slight and slow. Therefore, the time of 50% release (*T*_1/2_) of about 18 h implies the drug release from the controlled drug delivery system is controllable and sustains in the natural conditions (pH 7.4). Therefore, it is expected that the provided nanoplatforms are suitable for controlled transfer, accumulation in tumor area and protecting of drug molecules from biodegradability and plasma clearance. 

The release curve shows that in 48 h, more than 60 percent of the drug is released from the formulation and then the trend of release growth will be slow.

*Toxicity of Gemcitabine hydrochloride and nanoparticles*

In this study with the aim of in-*vivo* applications, nanoparticles of SPION-PLGA-PEG-Gem were also prepared and the results showed that addition of PEG to PLGA has no considerable effect on physical properties of nanoparticles (Table 4), but for *in-vitro* toxicity experiments, nanoparticles without PEG were used. 

The viability (%) of the cells under treatment by Gemcitabine (with concentration between 0.01 µM to 25 µM) was studied using 24 h and 48 h MTT assays. As it can be seen in Figure 5. the repeatability of the toxicity data in 48 h incubation is better than the 24 h incubation. Moreover, According to the slope of the release curve in the first 24 h and slowing the release curve growth after 48 h (Figure 4), it can be concluded that to assess the toxicity of the nanoparticles, incubation time of 48 h is more appropriate and therefore considered for evaluation of toxicity of nanoparticles.

After determining the range of treatment concentration of Gemcitabine, the MCF7 cells were treated by different formulations with the same procedure, in the range of concentrations equivalent to the concentrations (0.01 μM to 10.3 μM) of Gemcitabine (released in 48 h).

Figure 6 illustrates the survival of MCF-7 cells under various treatment groups by nanomolar equivalent concentrations of Gemcitabine with 48-hours incubation using MTT assay. This figure obviously shows the effectiveness of the treatment with SPION-PLGA-Gem compared to Gemcitabine and the other nanoparticles, especially at higher concentrations. 

The range of inhibitory concentration of 10% (IC10) for Gemcitabine is about 10 nM. The toxicity of the other formulations containing 10 nM Gemcitabine is relatively equivalent to the toxicity of 10 nM Gemcitabine. SPION-PLGA nanoparticles with very higher concentrations (equivalent to 25 μM of Gemcitabine in other formulations) have no considerable toxicity (less than 10% toxicity). This suggests that the use of SPION in different formulations has a great advantage for therapeutic applications (hyperthermia and radiosensitization) or diagnostic applications (as a contrast agent in MR and ultrasound imaging). According to Figure 6. treatment with polymeric nanoparticles considerably had better result than Gemcitabine. The survivals of cell treated by concentration of formulations that were equivalent to IC50 of Gemcitabine were listed in Table 7. The survivals were 26.5% and 32.7% for SPIO-PLGA-Gem and PLGA-Gem respectively (that were equivalent to DER of 1.89 and 1.53).As illustrated in Figure 7. statistical differences between these groups are significant (*p*-values of 0.006 and 0.015 for SPIO-PLGA-Gem and PLGA-Gem groups compared with Gemcitabine group). It means the good treatment efficiency of formulations for MCF7 cells.In this study nanoparticles of SPION-PLGA-PEG-Gem were also prepared and the results showed that addition of PEG to PLGA had no considerable effect on physical properties of nanoparticles (*Z*_ave _= 174.0 ± 8.0 nm, PDI = 0.08 ± 0.03 and zeta potential = 15 ± 1.6 mV) but for *in-vitro* experiments, nanoparticles without PEG were used.

## Conclusion

In the present study, a novel Gemcitabine loaded super paramagnetic PLGA-Iron Oxide multifunctional drag delivery system was developed. This optimization was designed based on applications such as radio sensitization, hyperthermia and ultrasound and MR imaging by contrast agents. By improving the issues related to the instability of Gemcitabine, an appropriate system was obtained for transfer and enhancement of antitumor activity of Gemcitabine.

Regarding to the studies on the use of SPIONs for MR and US imaging purposes, it seems that these nanoparticles are easily able to meet the goals in these modalities. However, the use of magnetic nanoparticles in hyperthermia will provide a better effectiveness of Gemcitabine in inhibition of tumor cells. 

Nanoparticles such as SPIO-PLGA-Gem that their efficiency have proved for diagnosis and treatment and drug delivery objectives, may be worthy as subjects for future studies. Future evaluations include *in-vitro* and *in-vivo* investigation of radiosensitization and other applications of these nanoparticles. 
